# The Effects of Using a Ramp and Elevator to Load and Unload Trailers on the Behavior and Physiology of Piglets

**DOI:** 10.3390/ani4030535

**Published:** 2014-09-11

**Authors:** John McGlone, Avi Sapkota

**Affiliations:** Animal and Food Sciences, Texas Tech University, Lubbock, TX 79409, USA; E-Mail: asapkota@purdue.edu

**Keywords:** elevator, piglets, ramp, welfare

## Abstract

**Simple Summary:**

Transport is a routine practice in the modern swine industry. Loading the pigs into trailers can be a novel and stressful experience for the animals. This study compared behaviors and physiological variables during and after loading using a ramp or elevator to determine which method is the least stressful to the pigs. Loading pigs by ramp appears to cause more stress than loading by elevator.

**Abstract:**

Transport is an inevitable process in the modern U.S. swine industry. The loading process is a novel and potentially stressful experience. This study uses behavior, heart rate and leukocyte counts to compare stress one hour before, during and after loading via ramp or elevator. Piglets were held in a home pen (control (CON)), walked up and down an aisle (handled (HAN)), or walked to a truck and loaded via elevator (ELE) or ramp (RAM). Sitting, feeding and blood parameters did not show a significant treatment by time effect (*p* > 0.05). Standing behavior did not differ between CON and HAN piglets nor between RAM and ELE piglets (*p* > 0.05); however, CON and HAN piglets stood more than RAM and ELE piglets during treatment (*p* < 0.05). After treatment, drinking behavior was increased in RAM piglets (*p* < 0.05). The heart rate of ELE piglets decreased 6.3% after treatment; whereas the heart rate of RAM piglets remained elevated 2.4% (*p* < 0.05). In terms of heart rate, loading by elevator appears to be less stressful than loading by ramp.

## 1. Introduction

In the majority of swine production systems in the U.S., piglets are farrowed at sow farms and transported to grower-finisher sites at weaning. When the piglets are to be transported, they are handled and moved from their home pens, walked along an aisle, then loaded into the trailer. Trailers used to transport these piglets are usually pot-bellied or straight-deck designs [1]. Metal, wooden or concrete ramps of various angles are commonly used to load the piglets from the aisle to the trailer. Additionally, more ramps may be used within the trailer to move piglets from one deck to another. Trailers in the European Union (EU) may be equipped with hydraulic lifts to move pigs to the desired deck of the trailer, preventing the pigs from having to walk up a sloped ramp [2].

Handling prior to transportation contributes to the stress of the event. Handling stressors may include being moved from the home pen, mixing of conspecifics, use of handling aides (prods, boards, paddles, flags) and distance moved. During periods of physical or psychological stress, the body responds with the activation of the hypothalamic-pituitary-adrenal (HPA) axis. This results in secretion of cortisol from the adrenal glands [3]. A study done by Dhabhar *et al*. showed that cortisol secretion in rats experiencing 1 hour of acute physical stress was associated with decreased lymphocyte count and increased neutrophil count [4].

Parrott *et al*. found no significant differences in heart rate and plasma cortisol in ewes loaded by elevator and ramp [5]. It can be surmised that pigs would respond in a similar fashion, but there is limited literature regarding the use of elevators and swine stress and welfare. Two studies have shown that there was no difference in muscle pH between elevator and ramp loaded pigs and, therefore, no effect of loading stress on meat quality [6,7]. Another study found that loading via elevator reduced heart rate compared to loading via ramp [8]. While improving meat quality is an important aspect of the pork production process, it is also important to focus on animal welfare. Currently, improving animal welfare is a major concern to both the swine industry and the public; increasing animal welfare may decrease transport losses, improve meat quality and decrease economic losses. This study was designed to compare the use of a ramp and an elevator to load and unload piglets and to determine which method is the least stressful to the animals.

## 2. Experimental Section

### 2.1. General

**Figure 1:**
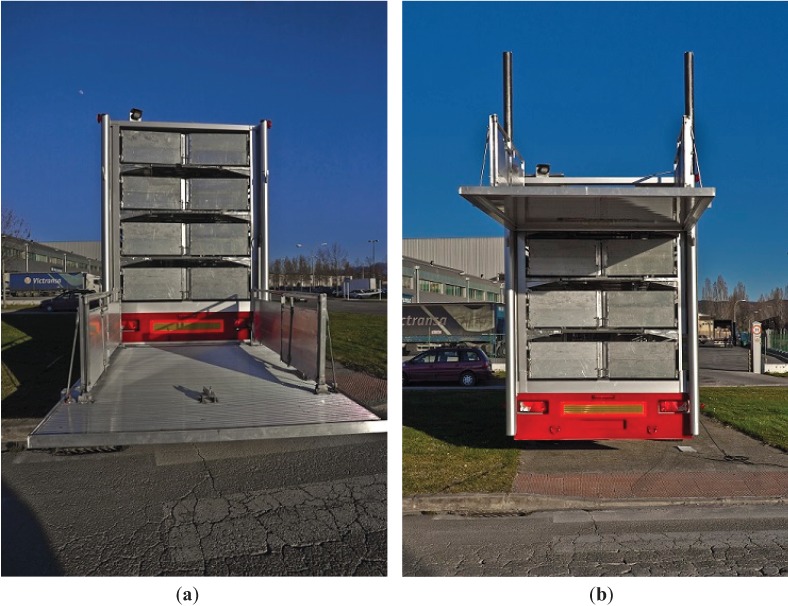
(**a**) Hydraulic lift elevator in the loading position. (**b**) Hydraulic lift elevator in the raised position.

This project was approved by the Texas Tech University Animal Care and Use Committee. All animals were housed in 2.1-m × 1.5-m pens with slatted floors at the Texas Tech Swine Research Farm near New Deal (Lubbock), Texas. Each pen contains 1 nipple waterer and a 3-hole feeder. Feed and water were provided *ad libitum* in the pens. Ventilation in the barn was via exhaust fan and ceiling louvers. A total of 120 weaned pigs of mixed commercial genetics, approximately 30 days of age, were used for this study. Pigs were randomly divided into 12 pens of 10 pigs each, 3 pens per treatment. Pigs were allowed to acclimatize to the new environment for 1 week before the study began. Four treatment groups were assigned: control (CON), handled (HAN), ramp (RAM) and elevator (ELE). CON piglets were not handled or moved from their pen. HAN pigs were moved out of the home pen, walked up and down the length of the aisle and then moved back into the home pen. HAN piglets were not held at the end of the aisle for any time period. RAM piglets were moved out of the home pen, walked down the aisle and then loaded onto the trailer using a ramp. ELE piglets were moved from their home pen, walked down the aisle and loaded onto the trailer using a hydraulic lift (Figure 1a,b). ELE and RAM piglets were held on the trailer (*i.e.*, not transported) for 1 hour and then moved back off the trailer via the treatment method and back to their home pens. Feed and water were available *ad libitum* in the home pens. Water was not available in the alley nor on the trailer. The movement of the pigs was facilitated by the use of sorting boards in accordance with the Transport Quality Assurance program guidelines [9]. Each treatment had 3 replications. Replications were performed on the same day, with at least 1 hour of rest between replications.

### 2.2. Design of the Aisle, Ramp and Elevator

Pigs in the HAN, ELE and RAM groups walked a distance of 36 to 46 m inside the building in a 1.2 m-wide aisle.

The ramp was metal, 0.8 m wide with cleats 0.3 m apart to reduce slipping and falling. The ramp chute was solid on the sides, up to 0.9 m high and partially open above that. For RAM piglets, the ramp was 4.6 m long set at a 20° angle.

ELE piglets were moved along the same aisle, across the ramp (now adjusted to 0°) and onto the elevator floor. The floor was elevated to the desired deck using a remote control.

Holding pens on the trailer were 2.4 m by 2.1 m in dimension.

### 2.3. Heart Rate Data Collection

Two randomly selected piglets from each group wore a jacket fitted with a heart rate monitor (Jacketed External Telemetry (JET), Data Science International, St. Paul, MN, USA) and were allowed to be as near as possible to their pen mates (when placed in the same pen, other pigs tended to chew at the jacket, altering the results and causing more stress to the pig), while the heart rate data were collected for 30 minutes. Therefore, heart rate data were collected using only two pigs out of the pen. Heart rate data were collected first. Then, the pigs went back to their home pen, had at least 1 hour of rest, and the whole group was rerun for behavior and blood sample collection. The pigs used for heart rate were not used for blood sample collection, as they already had exposure to handling and treatments.

### 2.4. Blood Sample Collection

Blood samples were collected via the right side jugular vein using a vacutainer with ethylene diamine-tetraacetic acid (EDTA). Two piglets from each pen were randomly selected for blood collection. Five to 7 mL of blood were drawn at each time. Blood samples were placed on ice and transported back to the laboratory at the Department of Animal and Food Sciences, Texas Tech University. Blood parameters were measured by Cell Dyne (IDEXX Laboratories, Inc., Westbrook, ME, USA) for differential blood leukocyte count. Values of interest include total white blood cell count (WBC), neutrophil count (N), lymphocyte count (L) and N:L ratio.

### 2.5. Behavior

All piglets were observed by the same individual, and behavior was categorized into one of the following: standing, sitting, lying, drinking or feeding. Each pen was scan sampled every 5 minutes to record behaviors. Behavior was recorded as a percentage of piglets expressing each behavior.

### 2.6. Timeline for Data Collection

Timeline for heart rate (HR); A–D refer to periods:

HR-AHR-BHR-CHR-DAcclimation near home penHR before treatment startedHR after pigs were walked on the aisle or loaded into trailer using ramp or elevatorHR after pigs were again walked on the aisle, unloaded from trailer using ramp or elevatorHR after pigs were back near to their home pen5 min10 min10 min 10 min5 min

Timeline for bleeding; A–D refer to blood sampling times:
BLEED-ABLEED-BBLEED-CBLEED-DBaseline, one hour before treatmentAt the start of treatmentImmediately after treatments were initiated After pigs were back in their home pen

Timeline for behavior (BEHAV); A–C refer to periods:
BEHAV-ABEHAV-BBEHAV-CBefore treatment startedDuring treatmentAfter treatment1 hour1 hour1 hour

For HAN pigs, behavior Time Period B includes some time immediately after the pigs were returned to the home pen. For instance, if a group of pigs took 10 minutes to be moved up the alley and back into the home pen, the next 50 minutes are included in the 1-hour observation for Time Period B.

### 2.7. Data Analyses

Each pen consisted of 9 or 10 piglets; therefore, the number of piglets displaying each behavior was converted to a percent of total pigs present in the pen. Heart rate data were summarized first as beats per minute (bpm) and then averaged to calculate as A, B, C and D, as mentioned above. Blood variables were also calculated as A, B, C and D. Behavior data were collected every 5 minutes and later averaged per hour as A, B and C, as mentioned above. The number of pigs showing a behavior were counted at first and later converted into the percent of pigs showing the behavior at a particular time point in each pen. The percent data were used for analysis. The data were analyzed using the PROC GLM statement in SAS 9.2 (SAS, 2010 SAS Inst., Inc., Cary, NC, USA). The model included treatment, pen within treatment, time and treatment × time effects as independent variables. The mean square of the pen within-treatment effect was used as the error term to determine the significance of the time and treatment by time interaction. Least square means were used to compare individual adjusted means.

## 3. Results and Discussion

### 3.1. General

There were significant treatment × time interactions for standing, drinking and heart rate (*p <* 0.01). Lying behavior tended to show a significant treatment × time interaction (*p* = 0.07). Other behaviors (sitting, lying and feeding) and blood parameters did not show a significant treatment by time effect (*p* > 0.05). All blood, heart rate and behavior results are summarized and displayed in Table 1.

**Table 1:** Summary of the percentage of piglets displaying behaviors, the values of blood parameters and the heart rates of pigs in response to treatments before, during and after treatments.

	Control				Handle				Elevator				Ramp					*p-*value (TRT × TIME)
**Period ^a^**	A	B	C	D	A	B	C	D	A	B	C	D	A	B	C	D	SE
**Sit ^b^ (%)**	1.4	1.6	1.9	-	2.1	3.7	0.6	-	2.6	3.7	0.8	-	2.8	2.0	1.7	-	0.82	0.3
**Stand ^b^ (%)**	37.8	37.2	21.4	-	31.3	24.7*	9.4	-	40.0	75.8*	23.2	-	37.0	93.9*	22.0	-	6.94	<0.01
**Lying ^b^ (%)**	45.8	48.9	66.7	-	57.2	64.1	84.1	-	47.3	17.2*	57.0	-	50.9	4.1*	59.2	-	8.48	0.07
**Drink ^b^ (%)**	14.7	11.7	8.9	-	9.1	7.5	5.4	-	9.8	0.0*	13.9	-	8.9	0.0*	15.8	-	1.96	<0.01
**Feed ^b^ (%)**	0.3	0.5	1.1	-	0.3	0.0	0.6	-	0.3	0.0	1.7	-	0.5	0.0	1.4	-	0.38	0.6
**WBC ^c ^(×10^3^/μL)**	20.8	23.4	23.7	23.2	18.2	19.4	20.6	21.8	19.3	23.6	26.1	23.8	19.4	25.9	29.0	26.8	1.16	0.16
**N ^d ^(×10^3^/μL )**	5.7	9.3	12.3	11.2	7.9	8.7	10.8	10.7	7.8	11.4	16.3	13.7	7.8	10.0	16.5	15.4	0.98	0.12
**L ^e ^(×10^3^/μL )**	12.6	11.9	9.3	10.4	9.2	9.6	8.7	7.2	10.5	10.9	8.5	7.3	10.7	14.6	10.9	9.9	0.94	0.38
**N:L RATIO ^f^**	0.5	0.8	1.3	1.1	0.9	0.9	1.3	1.7	0.8	1.1	1.9	2.3	0.7	0.7	1.5	1.6	0.23	0.58
**HR ^g ^(bpm)**	193.3	199.3	183.5	-	180.6*	183.2*	186.8	-	185.0	196.9	184.5	-	201.0	202.3	207.2*	-	3.1	0.02

* Indicates that treatment means are different than control means (*p* < 0.05); ^a^ A = one hour prior to treatment; B = immediately prior to treatment; C = immediately after treatment was initiated; D = one hour after treatment ended. ^b^ Behavior was observed every five minutes and averaged for each time period. Blank values indicate that data were not collected at that time period. ^c^ Total white blood cell count. ^d^ Neutrophil count. ^e^ Lymphocyte count. ^f^ Neutrophil:lymphocyte ratio. ^g^ Heart rate (HR): values were averaged over five minutes for Periods A and D and over 10 minutes for Periods B and C. Blank values indicate that data were not collected at that time period.

### 3.2. Behavior

**Figure 2:**
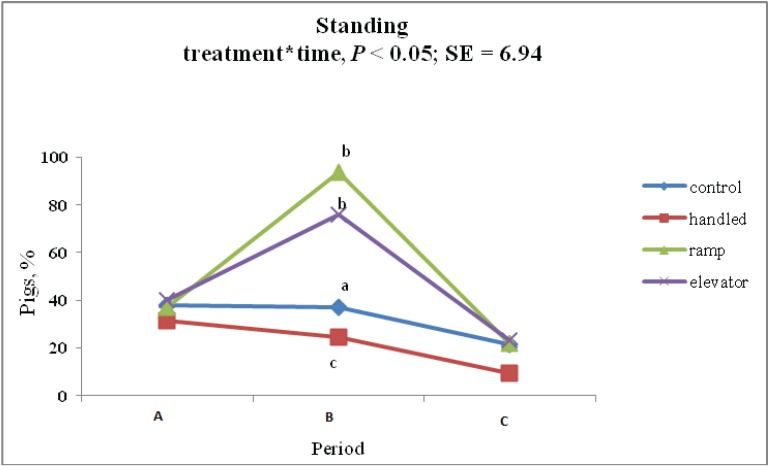
Percentage of pigs showing standing behavior an hour before (A), during (B) and after (C) each treatment (control, handling, ramp and elevator).

^a,b,c^ Different superscripts within a period represent differences in standing behavior.

A significant treatment × time interaction was observed for standing behavior during treatment (37.20 ± 6.94%, 24.70 ± 6.94%, 75.80 ± 6.94% and 93.90 ± 6.94% for CON, HAN, ELE and RAM, respectively; *p* < 0.01). Standing behavior did not differ among CON and HAN pigs (*p* > 0.05). ELE and RAM piglets showed greater frequency of standing behavior than did CON and HAN (*p* < 0.05); however, standing behavior among ELE and RAM piglets did not differ from each other during treatment. Figure 2 shows the percentage of piglets standing at each time point.

**Figure 3:**
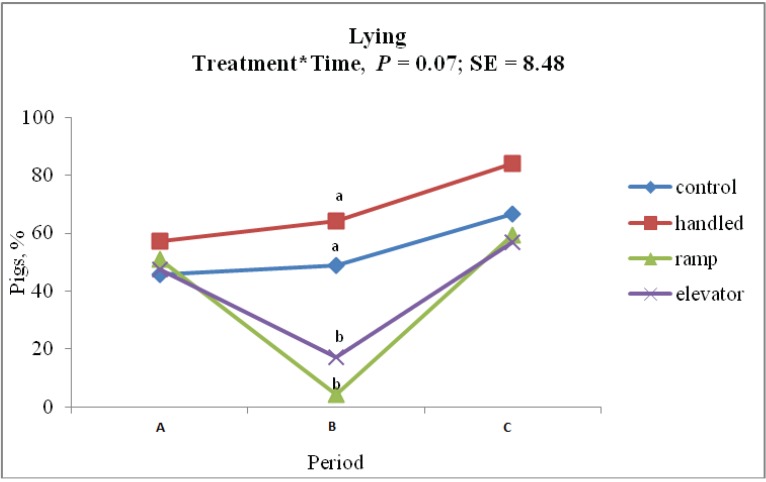
Percentage of pigs showing lying behavior an hour before (A), during (B) and after (C) each treatment (control, handling, ramp and elevator).

^a,b^ Different superscripts within a period represent differences in lying behavior.

There was a tendency towards a significant treatment × time effect for lying behavior (48.9 ± 8.48%, 64.1 ± 8.48%, 17.2 ± 8.48% and 4.1 ± 8.48% for CON, HAN, ELE and RAM, respectively; *p* = 0.07). The percentage of piglets displaying lying behavior during treatment was not different for CON and HAN piglets (*p* > 0.05); however, a lesser percentage of ELE and RAM piglets displayed lying behavior during treatment. The ELE and RAM piglets did not differ from each other for lying behavior during treatment (*p* > 0.05). Figure 3 shows the percentage of piglets lying at each time point.

**Figure 4:**
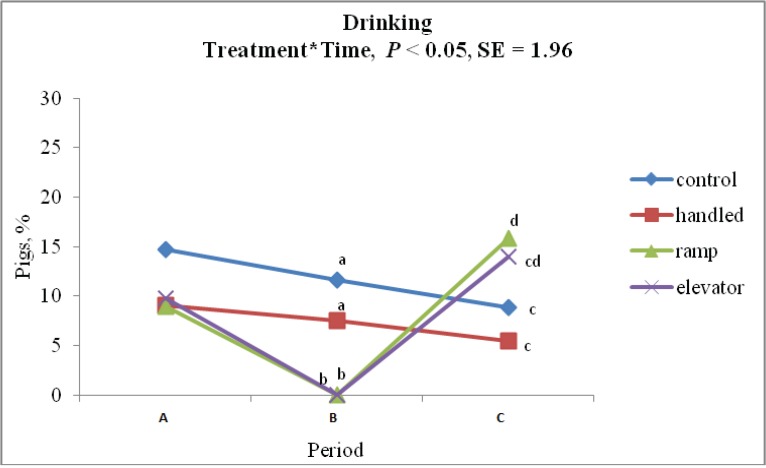
Percentage of pigs showing drinking behavior an hour before (A), during (B) and after (C) each treatment (control, handling, ramp and elevator).

^a,b,c,d^ Different superscripts within a period represent differences in drinking behavior.

There was a significant time × treatment interaction for drinking behavior (*p* < 0.01). During the time of treatment, the percentage of CON (11.70 ± 1.96%) and HAN (7.50 ± 1.96%) piglets showing drinking behavior did not differ (*p* > 0.05). ELE and RAM piglets did not show any drinking behavior during the time of treatment. When piglets were brought back to the home pen, drinking behavior was higher in RAM (15.80 ± 1.96%) piglets compared to CON (8.90 ± 1.96%) and HAN (5.40 ± 1.96%) piglets (*p* < 0.05). The percentage of ELE piglets showing drinking behavior after treatment (13.90 ± 1.96%) was not different from CON, HAN and RAM piglets. Figure 4 shows the percentage of piglets drinking at each time point.

### 3.3. Heart Rate

**Figure 5:**
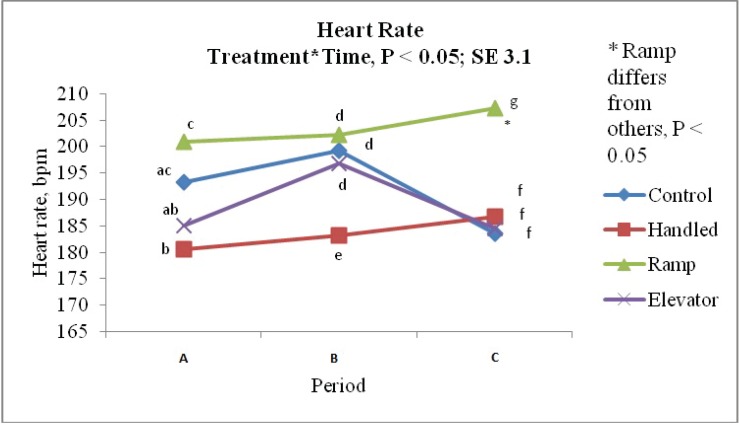
The heart rate (beats per min) of pigs an hour before, during and after each treatment (control, handling, ramp and elevator).

^a,b,c,d,e,f,g^ Different superscripts within a period represent differences in heart rate.

A significant treatment × time interaction was observed for heart rate (*p* = 0.02). The heart rate of ELE piglets decreased from 196.90 ± 3.10 bpm during treatment to 184.50 ± 3.10 bpm when piglets were placed back in the home pen (*p* = 0.01). The heart rate (bpm) of RAM piglets, on the other hand, increased from the “during” period (202.30 ± 3.10) to the “after” period (207.20 ± 3.10) (*p* > 0.05). Figure 5 shows the heart rate of piglets at each time point.

## 4. Conclusions

The behavior of pigs can vary before, during and after a stressful event. In addition, physiological measures, such as temperature, respiratory rate, heart rate, blood parameters and immune measures, are affected by stress [10,11]. Sitting, standing, lying, feeding and drinking behaviors before, during and after piglets were loaded into the trailer either using a ramp or elevator were compared with control and handled piglets. The number of piglets showing standing behavior inside the trailer did not differ between ELE and RAM piglets, but these numbers were greater than the numbers of CON and HAN piglets showing standing behavior at this time point. This shows that piglets were more restless in the trailer, which may be due to the novelty and stress of being moved. Feeding and drinking behavior were null when the piglets were in the trailer, because feed and water were unavailable in the trailer.

The heart rate of ELE pigs decreased after the pigs were brought back to the home pen; on the other hand, the heart rate of RAM pigs remained elevated. The heart rates of RAM and ELE piglets did not differ during treatment. The heart rate is considered an indicator of stress, and it may have been elevated in these treatment groups, due to the novelty of the experiences. An analysis of cortisol concentrations is warranted in future studies to support other physiological changes indicative of stress. Brown [12] reported a lowered heart rate, but an elevated cortisol concentration when a modular system was used to load and unload pigs, showing that pigs might still be stressed even though the heart rate is not elevated. They also reported little difference between ramp and elevator use, which is inconsistent with the findings of this study.

Even though the blood parameters did not differ between the ELE and RAM piglets, handling intensity and use of handling aides (prods, boards, *etc.*) can influence skin lesions (due to slips and falls) and meat quality, therefore affecting the overall welfare and economic impact. Correa [13] showed that handling intensity and handling aides used while loading can affect the stress response and meat quality of pigs. Ramp angle is another factor to be taken into consideration. Grandin [2] recommended 15° as a maximum angle for ramps used to load and unload pigs. Warriss [14] reported that time to ascend or descend did not differ significantly when the ramp angle was between 0° and 20°; however, it took longer for pigs to climb steeper ramps.

Pigs are susceptible to stress and may become stressed when faced with having to adapt to any changes in their regular environments or novelty [15]. Pigs are typically unaccustomed to handling, walking long distances and using ramps [16]. Pigs are moved from their home pens usually when they are moved to a new facility, such as to nursery pens after weaning, to growers’ buildings after the nursery and to processing plants when they reach market weight. Pigs are usually transported via trailer and are loaded and unloaded via ramps. Stress due to the novelty of these experiences can lead to increased transit losses [1,17,18]. The transport of pigs cannot be avoided in the current multi-site production systems utilized in the U.S. The results of the current study agree with the findings of previous studies [5,8]. After the event, the heart rate is lower in pigs loaded by elevator compared to that of pigs loaded by ramp. Using an elevator to load and unload pigs can potentially minimize stress to the animals, as the handlers do not have to handle the pigs as much as they do when using ramps. Nonetheless, both the ramp and the elevator are novel experiences for most pigs and result in some level of stress during loading.
